# Intelligent Control of a Sensor-Actuator System via Kernelized Least-Squares Policy Iteration

**DOI:** 10.3390/s120302632

**Published:** 2012-02-28

**Authors:** Bo Liu, Sanfeng Chen, Shuai Li, Yongsheng Liang

**Affiliations:** 1 Key Lab of Visual Media Processing and Transmission, Shenzhen Institute of Information Technology, Shenzhen, Guangdong 518029, China; E-Mail: liangys@sziit.com.cn; 2 Department of Computer Science, University of Massachusetts, Amherst, MA 01003, USA; E-Mail: boliu@cs.umass.edu; 3 Department of Electrical and Computer Engineering, Stevens Institute of Technology, Hoboken, NJ 07030, USA; E-Mail: lshuai@stevens.edu

**Keywords:** Markov Decision Process, sensor-actuator systems, random Projections, Kernelized Least Square Policy Iteration

## Abstract

In this paper a new framework, called Compressive Kernelized Reinforcement Learning (CKRL), for computing near-optimal policies in sequential decision making with uncertainty is proposed via incorporating the non-adaptive data-independent Random Projections and nonparametric Kernelized Least-squares Policy Iteration (KLSPI). Random Projections are a fast, non-adaptive dimensionality reduction framework in which high-dimensionality data is projected onto a random lower-dimension subspace via spherically random rotation and coordination sampling. KLSPI introduce kernel trick into the LSPI framework for Reinforcement Learning, often achieving faster convergence and providing automatic feature selection via various kernel sparsification approaches. In this approach, policies are computed in a low-dimensional subspace generated by projecting the high-dimensional features onto a set of random basis. We first show how Random Projections constitute an efficient sparsification technique and how our method often converges faster than regular LSPI, while at lower computational costs. Theoretical foundation underlying this approach is a fast approximation of Singular Value Decomposition (SVD). Finally, simulation results are exhibited on benchmark MDP domains, which confirm gains both in computation time and in performance in large feature spaces.

## 1. Introduction

This paper explores a technique called *compressive reinforcement learning*, analogous to recent work on compressed sensing, wherein approximation spaces are constructed by measurements representing random correlations with value functions. A Random Projections is a simple but elegant technique that has both a strong theoretical foundation and a wide range of applications including signal processing, medical image reconstruction, machine learning and data mining. Its theoretical foundation rests on the Johnson–Lindenstrauss lemma [1]: given a set of samples *S* in a high-dimensional feature space *R^n^*, if we construct an orthogonal projection of those sample points onto a random *d*-dimensional subspace, then if $d=O\left(\frac{\text{log}|S|}{{ɛ}^{2}}\right)$, the projection is Lipschitz; in other words, pairwise distances are preserved with high probability (*P* > 1/2) up to a small distortion factor of 1 ± *ɛ*. Intuitively, this process can be thought of as applying a random rotation to a high-dimensional manifold and then reading off the first *d* coordinates. Compared with other linear dimension reduction methods, like Principal Component Analysis (PCA), Factor Analysis (FA), *etc.*, Random Projections are data-independent, which significantly reduces the computational cost. In [2], Least-square temporal difference algorithm (LSTD) [3] is analyzed with approximation error analysis in finite-sample scenario. In [4], Random Projections are integrated with reinforcement learning algorithms that solve Markov Decision Processes (MDPs) in the context of least-squares temporal difference learning setting.

Kernelized reinforcement learning, as it is named, aims at bring the benefits of non-parametric kernel approaches to reinforcement learning algorithms family. A kernelized least-squares policy iteration (KLSPI) is proposed in [5] by replacing inner product via kernel in LSTD architecture. [6] follows similar style by introducing kernel approach into LSPE [7] framework. Other approaches, such as [8–10], seems to be more inspired from the Gaussian processes, where the covariance function is displaced with kernel function. Meanwhile, *L*_2_ regularization is also intensively studied in these Gaussian process driving approaches and also in [11]. In KLSPI, kernels are used as basis for efficient policy evaluation. A sparsification procedure based on approximate linear dependency (ALD) is performed for sparsification, which is an online, fast approximate version of PCA [12]. KLSPI reaches two progresses: One is better convergence both in reduced convergence time and in better convergence precision than regular LSPI, the other is automatic feature selection via ALD-based kernel sparsification. Therefore, the KLSPI algorithm provides a general RL method with generalization performance and convergence guarantee for large-scale MDP problems.

In this paper, a new framework, called Compressive Kernelized Reinforcement Learning (CKRL), for computing near-optimal policies in sequential decision making with uncertainty is proposed via incorporating the non-adaptive data-independent Random Projections and nonparametric Kernelized Least-squares Policy Iteration (KLSPI). One of the central ideas is that Random Projections are able to constitute an efficient sparsification technique and this brings about both faster convergence rate and lower computational costs than regular LSPI. Theoretical foundation underlying this approach is a fast approximation of Singular Value Decomposition (SVD). Experimental results also demonstrate that this approach enjoys the benefit of nonparametric approaches as well as alleviating the computation cost induced by non-adaptive random projections.

Here is a brief roadmap to the rest of the paper. In Section 2, background knowledge of the three major perspectives comprising this paper is introduced on compressed sensing and random projections, kernel regression and sparsification and approximate Markov Decision Processes algorithms. In Section 3, unified framework and overview of state-of-art kernelized reinforcement learning algorithm is given, with extensive analysis on both kernel sparsification and error decomposition analysis. In Sections 4 and 5, the algorithms of Compressive Kernelized Reinforcement Learning algorithm are proposed with intensive theoretical analysis in Section 5. Finally, experimental results are conducted in the context of various experimental settings on different benchmark domains to validate the effectiveness of the proposed approach in Section 6.

## 2. Background

### 2.1. Compressed Sensing and Random Projections

Let us first have a brief review of some important concepts and theorems that will be used in this paper.

**Lemma 1: (Restricted Isometry Property)** A *m* × *n* compression matrix *C* ∈ *R*^*m*×*n*^ satisfies the Restricted Isometry Property (RIP), (*k*, *ε*)-RIP, if it acts as a near-isometry with distortion factor *ε* over all *k*-sparse vectors, that is, for any *k*-sparse vector *x* ∈ *R^n^*, the following near-isometry property holds

(1) $$(1-\varepsilon ){\left\|x\right\|}_{2}\leq {\left\|\mathrm{Cx}\right\|}_{2}\leq (1+\varepsilon ){\left\|x\right\|}_{2}$$

Compressed Sensing has recently drawn attention as an efficient way of reducing variance at the cost of increasing bias within a tolerable extent. Random Projections (RP) can be viewed as a simple and elegant implementation of it. In Random Projections, high-dimensional data is projected onto a lower-dimensional subspace using a randomly generated compression matrix *C* whose columns have unit lengths. Each entry of *C* matrix *c_ij_* is draw from zero mean, unit variance distributions, which looks as if using random noise basis at the first glance, and exert a pairwise distance preserving projection that satisfies Johnson–Linderstrauss Lemma. Geometrically, RP is a simple geometric technique for reducing the dimensionality of a set of points in Euclidean space while approximately preserving pairwise distances with high probability *P* > 1/2 (w.h.p). If $d=O\left(\frac{\text{log}|S|}{{ɛ}^{2}}\right)$, the projection is Lipschitz, that is, pairwise distances are preserved w.h.p up to a distortion factor of 1 ± *ɛ*. From the sampling perspective, RP is performing coordinate sampling after a spherically random rotation to *R^n^* [13]. Intuitively, this process can be thought of as applying a spherical random rotation to a high-dimensional manifold and then reading off the first *d* coordinates. RP is computationally efficient because of its data-independence nature, yet sufficiently accurate for dimensionality reduction of high-dimensional data sets.

### 2.2. Kernel Regression, Regularization and Sparsification

Now we give a brief introduction of kernel, kernel matrix, kernel trick and kernel regression. A kernel is a symmetric function representing the similarity between two samples,

(2) $$k(x_{i},\;x_{j})=k(x_{j},\;x_{i})$$

Given sample set ${\left\{x_{i}\right\}}_{i=1}^{n}$, a kernel matrix *K* is a symmetric matrix with each entry *K_ij_* = *k* (*x_i_*, *x_j_*). If the kernel matrix *K* is not only symmetric but also positive semi-definite, Mercer’s theorem states that the kernel function can be interpreted as an inner product of nonlinear function *ϕ* (·, ·) between every pair of instances without explicitly knowing the nonlinear function’s form. Kernel trick is application of Mercer’s theorem to enrich the expressiveness of the feature space without manually adding more features. Geometrically, kernelization is a way of obtaining linearity by practising a high-dimensional embedding through a nonlinear mapping: First, an inner product is introduced by defining the “nonlinear” kernel, then the whole space is embedded into the high-dimensional kernel space with inner-product preserving, and finally linearity is obtained in this high-dimensional kernel space.

Kernel regression [14], also called the Nadaraya–Watson model, is a kernelized form of linear least-square regression [15]. Given the linear model *t* = Φ*w* + *ε*, the sum-of-squares error function without *L*_2_ regularization term is given by $J(w)=\frac{1}{2}\sum_{i=1}^{n}{\left\{w^{T}\;\phi \;(x_{i})-t_{i}\right\}}^{2}$. Introduce the representation of *w* = Φ*^T^* *a*, and Gram matrix *K* = ΦΦ*^T^*, we have $J=\frac{1}{2}a^{T}\;K\;\mathrm{Ka}-a^{T}\;\mathrm{Kt}+\frac{1}{2}t^{T}\;t$. So the kernel regression for the linear model is

(3) $$y(x)=k{\left(x\right)}^{T}K^{-1}\;t$$

where *t* represents the target values of the sample points, and *k*(*x*) is a column vector where *k_i_* (*x*) = *k* (*x*, *x_i_*). The problem with this method is that there is no guarantee that the kernel matrix is invertible. The most common remedy for this problem is to introduce ridge regression/*L*_2_ regularization into this kernelized version, adding regularization term *λI* to the diagonal so that *K* will be invertible. Thus (3) would become

(4) $$y(x)=k{\left(x\right)}^{T}\;\;{\left(K+\lambda I\right)}^{-1}\;t$$

Another idea is to reduce the dimension of the kernel matrix *K* while preserve its rank so that it is more likely to be nonsingular as its dimension decreases. ALD method can be considered as a tentative approach in this category. Another approach is to try to practise the pairwise-distance preserving compression, e.g., using Random Projections for matrix compression. We hereby give a brief review of Compressed Linear Regression by [16]. Suppose we have a randomly generated compression matrix *C* = *C_m,n_*. Firstly we introduce compression matrix *C_d,n_* to linear model, so there is

Ct=CΦw+Cε

and we define

$$t_{C}=\mathrm{Ct},\;\Phi_{C}=C\Phi$$

The sum-of-squares error function without *L*_2_ regularization term is given by

$$J_{C}\;(w)=\frac{1}{2}\sum_{i=1}^{n}{\left\{{\left(\mathrm{Cw}\right)}^{T}\;\;(C\phi (x_{i}))-t_{i}\right\}}^{2}$$

Introduce the representation of $w_{C}=\Phi_{C}^{T}\;a_{C}$, and Gram matrix $K_{C}=\Phi_{C}\;\Phi_{C}^{T}={\mathrm{CKC}}^{T}$, we have

$$J_{C}=\frac{1}{2}a_{C}^{T}\;K_{C}\;K_{C}\;a_{C}-a_{C}^{T}\;K_{C}\;t_{C}+\frac{1}{2}t_{C}^{T}\;t_{C}$$

with

$$a_{C}=K_{C}^{-1}\;t_{C}$$

In the end there is kernel regression for this compressed linear model of

(5) $$y(x)={\left(\mathrm{Ck}(x)\right)}^{T}\;\;{\left({\mathrm{CKC}}^{T}\right)}^{-1}\;\;(\mathrm{Ct})$$

We now give proof that the compressed kernel matrix *CKC^T^* is nonsingular whenever *rank*(*K*) ≥ *d*.

**Theorem 1**: If the kernel matrix *K* satisfies *rank*(*K*) ≥ *d*, the compressed kernel *CKC^T^* will be nonsingular, *i.e.*, *rank*(*CKC^T^*) = *d*, where *C* is the randomly generated compression matrix.

**Proof**:

If *rank*(*K*) ≥ *d*, there exists a sequence {*i*_*r*1_, *i*_*r*2_, ⋯, *i_rd_*} and {*i*_*c*1_, *i*_*c*2_, ⋯, *i_cd_*} such that the sub-matrix *K_sub_* which are drawn from *K* with rows of index {*i*_*r*1_, *i*_*r*2_, ⋯, *i_rd_*}, and columns of index {*i*_*c*1_, *i*_*c*2_, ⋯, *i_cd_*} such that *rank* (*K_sub_*) = *d* Next draw arbitrary *d* rows and columns from *C*, which form square matrix *C_sub_* such that

(6) $$\mathrm{rank}\;(C_{\mathrm{sub}}\;K_{\mathrm{sub}}\;C_{\mathrm{sub}}^{T})\leq \mathrm{rank}\;({\mathrm{CKC}}^{T})\leq d$$

Since each column of *C* is approximately orthogonal, so is *C_sub_*. Also since the rank of a matrix is invariant by left-multiplying a full column-rank matrix or right-multiplying a full row-rank matrix, we have $\mathrm{rank}\;(C_{\mathrm{sub}}\;K_{\mathrm{sub}}\;C_{\mathrm{sub}}^{T})=d$ So combining Equation (6) and above equations, altogether we have *rank* (*CKC^T^*) = *d*. This explains why the compressed kernel matrix *CKC^T^* is more prone to be nonsingular compared with the original kernel matrix *K*, which provides an alternative way besides ridge regression to handle *K* if it is ill-conditioned.

### 2.3. Approximate Solutions of Markov Decision Processes in Large Feature Space

A *Markov Decision Process* (MDP) [17] is defined by the tuple (*S*, *A*, $P_{{\mathrm{ss}}^{\prime }}^{a}$, *R*, *γ*), comprised of a set of states *S*, a set of (possibly state-dependent) actions *A* (*A_s_*), a dynamical system model comprised of the transition probabilities $P_{{\mathrm{ss}}^{\prime }}^{a}$ specifying the probability of transition to state *s*′ from state *s* under action *a*, and a reward model *R*. A policy *π*: *S* → *A* is a deterministic mapping from states to actions. Associated with each policy *π* is a value function *υ^π^*, which is a fixed point of the Bellman equation:

(7) $$\upsilon^{\pi }=T^{\pi }(\upsilon^{\pi })=R^{\pi }+\gamma P^{\pi }\;\upsilon^{\pi }$$

where 0 ≤ *γ* < 1 is a discount factor. In what follows, we often drop the dependence of *υ^π^* on *π*, for notational simplicity. When the set of states *S* is large, it is often necessary to approximate the value function *v* using a set of basis functions (e.g., polynomials, radial basis functions, wavelets *etc.*). In linear value function approximation, a value function is assumed to lie in the linear span of a basis function matrix Φ of dimension *S* × *k*, where we assume that *k* ≪ |*S*|. Hence, *υ* ≈ *υ̂* = Φ*w*. The vector space of all value functions is a normed inner product space, where the “length” of any value function *f* can be measured with respect to a weighted norm *ξ* as

(8) $${\left\|f\right\|}^{2}=\sum_{s}\xi (s)\;f^{2}\;(s)=f^{\prime }\;\Xi \;f$$

where Ξ is a diagonal matrix whose entries are given by *ξ*. It is often the case that *ξ* is selected to be the invariant distribution of the Markov chain induced by a policy *π*, where the distribution is assumed to be ergodic. For example, one approach to approximately solving the Bellman equation is the fixed point (FP) solution, often referred to as the TD approach, which is defined as finding a set of weights *w_FP_* such that

(9) $$w_{\mathrm{FP}}=\underset{w}{\text{arg min}}{||\Pi^{\Phi }\;T(\Phi w)-\Phi w||}_{\xi }^{2}$$

where Π^Φ^ is the orthogonal projection onto the column space of Φ, given by

$$\Pi^{\Phi }=\Phi {\left(\Phi^{T}\Xi \Phi \right)}^{-1}\Phi^{T}\Xi$$

Fixed point solutions, therefore, look for a fixed point of the *combined* projected back-up operator Π^Φ^*T*. Bellman residual methods, in contrast, find a set of weights *w_BR_* under which the difference between the approximated value function and the back-up approximated value function *T*(Φ*w*) is minimized. That is,

(10) $$w_{\mathrm{BR}}=\underset{w}{\text{arg min}}{||T(\Phi w)-\Phi w||}_{\xi }^{2}$$

Least-squares policy iteration (LSPI) is a well-known reinforcement learning method that can be combined with either the FP or BR projections to find the optimal (approximate) value function that lies in the linear space spanned by Φ. LSPI can be viewed as an approximate policy iteration method that combines regular policy iteration with least-squares approximation. Regression in large feature space often gives rise to overfitting in high-dimensional learning task where the number of features is larger than the number of samples. Up to now, the most prevailing method in large feature space is *L*_1_-based feature selection method, *i.e.*, LARS, LASSO, *etc.* Compressed Sensing and Random Projections provide another path to tackle this problem as an alternative to *L*_1_-regularization, e.g., the work presented in [18], which provides bounds on Compressed Least-squares (CLSR) errors compared with errors in the initial space. The main conclusion is that the estimation error is reduced as a result of alleviation of overfitting, but the approximation error is increased due to the subspace assumption. In a nutshell, CLSR method first select a random subspace and performs an empirical risk minimization in the compressed domain. In the compressed domain the estimation error is reduced at the cost of a “controlled” additional approximation error. It is also proved that using CLSR, the estimation error is bounded by $O\left(\frac{\text{log}\;n}{\sqrt{n}}\right)$. It is an interesting alternative to *L*_1_-regularization methods.

In Reinforcement Learning, it is indeed a huge computational challenge for LSPI to work with large amount of features. Besides heavy computation costs, another concern is learning performance, since a lot of training data is needed for large feature spaces. The third concern is data efficiency. Often the key to data-efficiency is sample reusage, *i.e.*, samples are not used only once (as in Q-learning), but for multiple times instead. Since samples would be scarce in the large feature space, data reusage is of critical importance.

Corresponding to the methods mentioned above, introducing *L*_1_ based method into LSPI, which is called LARS-TD [19], provides an effective way for *L*_1_ regularization and feature selection. Another way is to do feature compression with Random Projections as an alternative of feature selection, as proposed in [2], which is the application of CLRS in reinforcement learning. The third way is to implement the kernel trick in LSPI, e.g., kernel-based LSPI (KLSPI). Generally, the complexity of kernelized methods scales well with the feature dimension, but the bad news is that the complexity now depends on the number of data instead. Kernel sparsification, therefore, plays a critical role in the performance of KLSPI here. In [5], a kernel sparsification method called ALD originated from [12] on kernelized recursive Least-squares regression is implemented.

## 3. Kernelized Least-Squares Policy Iteration with Regularization

### 3.1. Kernelized Least-squares Policy Iteration

Nonparametric approximators have been combined with a number of algorithms for approximate policy evaluation. For instance, kernel-based approximators are combined with LSTD and LSTD-Q by [5,11], and with LSPE-Q by [6]. Document [10] used the related framework of Gaussian processes to approximate value functions in policy evaluation. Document [15] showed that, in fact, the algorithms in [8,10,12], are identically the same on condition that the same samples and the same kernel function are used. A kernel-based approximator can be seen as linearly parameterized if all the samples are known in advance. In certain cases, this property can be exploited to extend the theoretical guarantees about approximate policy evaluation from the parametric case to the nonparametric case [5]. Document [11] provided performance guarantees for their kernel-based LSTD-Q variant for the case when only a finite number of samples is available.

An important concern in the nonparametric case is controlling the complexity of the approximator. Computational burden, which is often the curse of nonparametric approximators in real applications of kernel-based methods and Gaussian processes, grows with the number of samples considered. Many of the approaches mentioned above employ various kernel sparsification techniques to limit sample complexity, *i.e.*, the number of samples that contribute to the solution ([5,12]).

Kernel-based LSPI introduces the kernel trick into least-square temporal difference learning to derive a kernelized version of LSTD. In KLSPI, kernels are used as basis in LSPI framework for efficient policy evaluation. A generalized kernelized model-based LSTD framework is presented in [15], where kernelized least-squares policy iteration is reduced to a more general framework of kernel regression in Reinforcement Learning. Kernel regression of the reward model and transition model can be depicted as follows, respectively.

Let us define *K*′ = *PK*, where

(11) $$K_{\mathrm{ij}}^{\prime }=E\;[k(s_{i}^{\prime },\;s_{j})]$$

We would like to note that unlike *K*, *K*′ is not symmetric. *K*′ can be thought of kernelized model of transition matrix *P*. The kernel regression of the reward model is

(12) $$\hat{R}\;(s)=k\;{\left(s\right)}^{T}\;\;K^{-1}\;R$$

The kernel regression of the transition model is

(13) $$\hat{k}\;(s)=k\;{\left(s\right)}^{T}\;K^{-1}\;K^{\prime }$$

Finally, the kernel regression of value function is

(14) $$V\;(s)=k\;{\left(s\right)}^{T}\;{\left(K-\gamma K^{\prime }\right)}^{-1}\;R$$

When the samples (*s_i_*, *a_i_*, *r_i_*, *s*′*_i_*, *π* (*s*′*_i_*)) are drawn from an identical trajectory, we have

(15) $$s_{i}^{\prime }=s_{i+1}$$

then

(16) $$\hat{V}\;(s)=k\;{\left(s\right)}^{T}\;{\left(K(I-\gamma G)K\right)}^{-1}\;\mathrm{Kr}$$

where *G* is a nil-potent upper shift matrix

(17) $$G=\left[\begin{array}{cc} {\vec{0}}^{T} & I_{n-1}\\ 0 & \vec{0} \end{array}\right],\;\vec{0}={\left[\begin{array}{ccc} 0, & \cdots , & 0 \end{array}\right]}_{1,n-1}$$

### 3.2. Regularization on Kernelized Reinforcement Learning Algorithms

How to successfully implement kernelized reinforcement learning algorithm involves various sparsification and regularization method. There are two main questions concerning the regularization. The first question is “How to regularize?”. To answer this question, there are several frameworks of *L*_2_-regularized kernelized LSTD, which can be roughly divided into three categories to the best of our knowledge. The first is adding regularization term to the kernel regression model of (12) and (13), respectively, *i.e.*,

(18) $$\begin{array}{l} \hat{R}\;(s)=k\;{\left(s\right)}^{T}\;{\left(K+\Sigma_{R}\right)}^{-1}\;R\\ \hat{k}\;(s)=k\;{\left(s\right)}^{T}\;{\left(K+\Sigma_{P}\right)}^{-1}\;K^{\prime } \end{array}$$

where Σ*_R_* and Σ*_P_* are *L*_2_-based regularization terms of reward model and transition model, respectively. Further discussions regarding the corresponding value function formulation and relations between the two regularization term Σ*_R_*, Σ*_P_* is in [15]. Gaussian Process Temporal Difference learning (GPTD) is also of this category.

Another category of regularized kernelized LSTD is in [11], in which a kernel matrix of size 2*n* is constructed, where *n* is the number of samples, which uses kernel values between pairs of next state. The authors have shown that the algorithm can be used efficiently when the value function approximation lie in a reproducing kernel Hilbert space (RKHS). They also developed finite sample error bound for the regularized algorithm.

**Lemma 2** [15]: The KLSPI value function is equivalent to the unregularized model-based value function given the same trajectories.

**Proof**: We give a sketch proof here which extends mainly three steps. In a same trajectory, *s*′*_i_* = *s_i_*_+1_, so we have *K*′ = *GK*. Secondly using *K*′ = *GK* we can have

(19) $$K(I-\gamma G)K=\mathrm{KK}-\gamma {\mathrm{KK}}^{\prime }$$

Combining Equations (19) and (16) gives

(20) $$\begin{array}{lll} \hat{V}\;(s) & = & k\;{\left(s\right)}^{T}\;{\left(K(I-\gamma G)K\right)}^{-1}\;\mathrm{Kr}\\ & = & k\;{\left(s\right)}^{T}\;{\left(\mathrm{KK}-\gamma {\mathrm{KK}}^{\prime }\right)}^{-1}\;\mathrm{Kr}\\ & = & k\;{\left(s\right)}^{T}\;{\left(K-\gamma K^{\prime }\right)}^{-1}\;r=V\;(s) \end{array}$$

The third sparsification technique is aiming at reducing sample complexity in streaming data, which renders it applicable in real applications. A sparsification procedure based on approximate linear dependency (ALD) can be performed, which is an online, fast approximate version of PCA [12]. ALD reaches two progresses. The first is better convergence both in reduced convergence time and in better convergence precision than regular LSPI. The other advantage is automatic feature selection using ALD-based kernel sparsification. Therefore, KLSPI provides a general RL method with generalization performance and convergence guarantee for large-scale MDPs.

Except the first question on how to implement to regularization, another question, both palpable and profound, is when to implement regularization. Generally there are two possible sparsification schemes which differ in when to practise sparsification: *preprocessing* and *postprocessing*. Preprocessing is to *directly* compress the feature space by feature selection. It then learns a basis of the sample (*s_i_*, *a_i_*) from the compressed feature space, and then use it in LSPI. In this case, it is equivalent to compressing the feature set in advance, and the same feature vector is used at every iteration for a new sample. The ALD sparsification is a typical method of this approach. In this method, each sample in the compressed dictionary spans a feature, and ALD is aiming at compressing this dictionary so that the number of elements in the dictionary is much smaller compared with the number of the whole sample set. In the ALD approach, a subset of samples *x̃*_1_, ..., *x̃_d_* is constructed in order to avoid redundant information. The dictionary is used such that *ϕ*(*x̃*_1_), ..., *ϕ*(*x̃_d_*) spans approximately *ϕ*(*x*_1_), ..., *ϕ*(*x_n_*), while being of minimal size. So the sparsification is actually in the feature space and can be counted as doing feature selection via abandoning redundant samples in forming the dictionary. KLSPI is an adaptation of this idea into LSTD framework. In [12], ALD is a kind of feature selection method, and can be considered as an online approximate algorithm of PCA, while at much reduced computational cost.

The postprocessing scheme is to *indirectly* adapt sparsification to LSTD, which is to learn a high-dimensional basis *k̃* (*s_i_*) of the sample (*s_i_*, *a_i_*, *r_i_*, *s*′*_i_*, *π* (*s*′*_i_*)) first, then use some technique for sparsification, and use the sparsified basis *k* (*s_i_*) in policy iteration to generate the new policy. In this case, one needs to recompute a basis at each iteration of LSPI since different feature vectors are used at each iteration, as *π* (*s*′) depends on the current policy. The *A*, *b* matrix is computed as follows:

(21) $$\begin{array}{lll} A & = & \sum_{i=1}^{n}k\;(s_{i})\;{\left(k\;(s_{i})-k\;(s_{i}^{\prime })\right)}^{T}\\ b & = & \sum_{i=1}^{n}k\;(s_{i})\;r_{i} \end{array}$$

### 3.3. Error Decomposition Analysis

Let us move to introduce the Bellman error of the kernelized value function, which is the one-step temporal difference error,

(22) $$\begin{array}{lll} \mathrm{BE}\;(\hat{V}) & = & R+\gamma P\hat{V}-\hat{V}\\ \mathrm{KBE}(\hat{V}) & = & R+\gamma P𝒦w-𝒦w \end{array}$$

**Figure 1.**
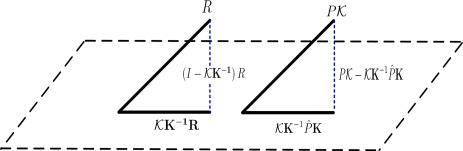
Illustration of Kernelized Bellman Error Decomposition.

According to [15], the Bellman error can be decomposed into two parts, which are not orthogonal to each other, the reward error and the transition error, which reflects the approximation error of the reward vector *R* and the transition matrix *P*, respectively. The geometric illustration can be seen in Figure 1, which is the kernelized version of Figure 1 in [20]. The Bellman error decomposition equation is as sequel,

(23) $$\begin{array}{lll} \mathrm{BE}\;(\hat{V}) & = & \Delta_{R}+\gamma \Delta_{\Phi }w_{\Phi }\\ \mathrm{KBE}\;(\hat{V}) & = & \Delta_{R}+\gamma \Delta_{K^{\prime }}w \end{array}$$

## 4. Algorithm Design

The General Framework of KLSPI with sparsification is described in Algorithm 1.

## 5. Theoretical Analysis

We use subtitle *C* to stand for compressed version, *K* for kernelized version, *CK* for kernelized LSTD-RP, and *KC* for compressed KLSTD. In the following, *n* stands for the number of samples, *D* stands for the dimension of full dimensional space, and *d* stands for the dimension of compressed dimensional space. In kernel regression, we have *D* = *n*.

**Algorithm 1.** General Framework of KLSPI.

***Input:***
• A sample data set (*s_i_*, *a_i_*, *r_i_*, *s*′*_i_*)
• A kernel function *k* (·, ·)
***Output:***
• policy *π*(*t*)
*Pre-processing* Sparsification to generate compact *Dic*
**REPEAT:**
**Policy Evaluation**:
Compute *k̃* (*s_i_*) based on *Dic*.
* Post-processing* Sparsification of *k̃* (*s_i_*) to generate *k* (*s_i_*)
Compute *A*, *b*
Compute solution *w* = *A*^−1^*b*, *V̂* (*s_i_*) = *k* (*s_i_*)*^T^ w*
**Policy Improvement:**
Compute Policy *π*(*t*)
**ENDLOOP**

Here is using ALD as an optional pre-processing sparsification step and LSTD-RP as a post-processing sparsification step.

**Algorithm 2.** KLSPI with Random Projections.

***Input:***
• A sample data set (*s_i_*, *a_i_*, *r_i_*, *s*′*_i_*)
• A kernel function *k* (·, ·)
• A compression dimension *d*
***Output:***
• policy *π*(*t*)
Use ALD to generate compact *Dic* (optional)
**REPEAT:**
**Policy Evaluation**:
Compute *k̃* (*s_i_*) based on *Dic*.
Construct compression matrix *C*.
*k* (*s_i_*) = *Ck̃* (*s_i_*)
Compute *A*, *b*
Compute solution *w* = *A*^−1^*b*, *V̂* (*s_i_*) = *k* (*s_i_*)*^T^w*
**Policy Improvement:**
Compute Policy *π*(*t*)
**ENDLOOP**

### 5.1. Random Projections is Approximate SVD

We have to be careful to extend any conclusion of linear projection to Random Projections since there is no guarantee that the Random Projections matrix is a projection operator, because *C* is generally not orthogonal (note that Random Projections is spherically random rotation plus coordinate sampling). A linear mapping can cause significant distortions in the data set if *C* is not orthogonal. Orthogonalizing *C* is, however, usually very computationally expensive and thus does not justify its cost. Instead, we can rely on a result in [21], that is, in a high-dimensional space, there exists a much larger number of ***almost*** orthogonal than orthogonal directions. Thus vectors having random directions might be sufficiently close to orthogonal, and equivalently *C^T^ C* ≈ *I*, where *I* is the identity matrix, and according to experimental experience [21], the mean squared difference between *C^T^ C* and an identity matrix was about $\frac{1}{k}$ per element. A lemma in [22] can be used to prove this.

**Theorem 2** Suppose that A is a real *m* × *n* matrix. Select a target rank *k* ≥ 2 and an oversampling parameter *p* ≥ 2, where *k* + *p* ≤ min {*m*, *n*}. Execute the proto-algorithm with a standard Gaussian test matrix to obtain an *m* × (*k* + *p*) matrix *Q* with orthonormal columns. Then the expectation of approximation error is bounded

$$E[||A-{\mathrm{CC}}^{T}\;A||]\leq \left[1+\frac{4\sqrt{k+p}}{p-1}\sqrt{\text{min}\{m,n\}}\right]\sigma_{k+1}$$

**Proposition 1**: For compression matrix *C*, the following holds:

1. Each column of *C* is approximately orthogonal.
2. *C^T^C* formulates an approximately identity matrix, *i.e.*, *C^T^ C* ≈ *I*.

Low rank matrix approximation is important in a wide variety of scientific applications including statistics, signal processing, machine learning, *etc.* Principal Component Analysis, as a unsupervised dimensionality reduction method, is the optimal solution when the target cost function is the sum of the mean square reconstruction error. The general idea of PCA, kernel PCA and ALD is to project the entire ambient feature space onto a lower dimensional manifold spanned by the topmost eigenvectors of the sample covariance matrix in feature space corresponding to the leading eigenvalues.

The problem formulation is trying to find a low rank matrix *X* to minimize the approximation error of ||*A* − *X*||. If we limit the minimizer to the form of *X* = *CC^T^ A*, the problem formulation of this family would be divided into *fixed-precision approximation* problem and *fixed-rank approximation* problem [22].

For the *fixed-precision approximation* problem, suppose we are given a *m* × *n* matrix *A* and a positive error tolerance *ε*. The task is to find a compression *d* × *m* matrix *C* with minimal *d* = *d* (*ε*) orthonormal column such that

$$d=\underset{C}{\text{min}}\;(\mathrm{rank}(C)),\;s.t.\;\;\;||A-C^{T}\;\mathrm{CA}||\;\leq \;\varepsilon$$

where ||·|| denotes the *L*_2_ norm. The range of *C* is a *d*–dimensional subspace that captures most of the actions of A, and *d* is desired to be as small as possible.

For *fixed-rank approximation* problem, given a matrix A, a predefined compression rank *d*, the problem is to find matrix *C* with orthonormal columns such that the approximation error *ε* = *ε* (*d*) is minimized.

$$\varepsilon =\underset{C}{\text{min}}\;(||A-C^{T}\;\mathrm{CA}||),\;s.t.\;\;\;\mathrm{rank}({\mathrm{CC}}^{T}\;A)\leq d$$

The singular value decomposition gives an optimal solution to the *fixed-precision problem*, *i.e.*, given a matrix A, a target compression rank *d*, the problem is to find

$$\underset{\mathrm{rank}(X)\leq d}{\text{min}}||A-X||,s.t.\;\;\;X=C^{T}\;\mathrm{CA}$$

The optimal solution is to construct the minimizer where the columns of *C* and the topmost *d* dominant left singular vectors of A, specifically, *X* = *USV^T^*, where *U_d_* is of size *n* × *d* and contains these *d* singular vectors, namely,

$$X^{\mathrm{SV}\;D}=U_{d}^{T}\;X$$

Based on Proposition 1, we can see that Random Projections can be considered as a fast, data-dependent approximate SVD which approximately preserves most of the actions of *A* in the compressed *d*-dimensional subspace.

### 5.2. LSTD with Random Projections

First let us have a brief review of LSTD-RP. In LSPI-RP, there is

(24) $$\begin{array}{lll} A_{C} & = & {\left(\Phi C^{T}\right)}^{T}\;\;(I-\gamma P)\;(\Phi C^{T})=C\;\Phi^{T}\;\;(\Phi -\gamma \Phi^{\prime })\;C^{T}=C\;{\mathrm{AC}}^{T}\\ b_{C} & = & {\left(\Phi C^{T}\right)}^{T}\;R=\mathrm{Cb} \end{array}$$

where *A*, *b* is defined in [23],

(25) $$\begin{array}{lll} A & = & \Phi^{T}\;(\Phi -\gamma \Phi^{\prime })=\sum_{i=1}^{n}\phi \;(s_{i})\;{\left(\phi \;(s_{i})-\phi \;(s_{i}^{\prime })\right)}^{T}\\ b & = & \Phi^{T}\;R=\sum_{i=1}^{n}\phi \;(s_{i})r_{i} \end{array}$$

Then the value function *V̂_C_* (*s*) is represented as

(26) $$\begin{array}{lll} {\hat{V}}_{C}\;(s) & = & {\left(C\phi \;(s)\right)}^{T}\;w\\ & = & {\left(C\phi \;(s)\right)}^{T}\;A_{C}^{-1}\;b_{C}\\ & = & {\left(C\phi \;(s)\right)}^{T}\;{\left(C\Phi^{T}\;(\Phi -\gamma \Phi^{\prime })\;C^{T}\right)}^{-1}\;C\Phi^{T}\;R \end{array}$$

### 5.3. Kernelized LSTD with Random Projections

Next we will prove the equivalence of the compressed Kernel-based LSTD with kernelized LSTD-RP, *i.e.*, if we perform Compressed Linear Regression to the sample set, and practise Kernel-based LSTD based on the compressed kernel matrix, this would be identical to the performance of the kernelized version of LSTD-RP for feature compression.

**Theorem 3**: The solution of the kernelized LSTD-RP is the approximate solution of the compressed kernel-based least-square temporal difference learning algorithm, namely,

(27) $$\begin{array}{lll} \hat{V}\;(s) & = & {\left(\mathrm{Ck}\;(s)\right)}^{T}\;{\left(C(K-\gamma K^{\prime })\;C^{T}\right)}^{-1}\;(\mathrm{CR})\\ & \approx & {\left(\mathrm{Ck}\;(s)\right)}^{T}\;{\left(\mathrm{CK}\;(K-\gamma K^{\prime })\;C^{T}\right)}^{-1}\;\mathrm{CKR}\\ & \approx & {\left(\mathrm{Ck}\;(s)\right)}^{T}\;{\left(CK^{\prime }(K-\gamma K^{\prime })\;C^{T}\right)}^{-1}\;CK^{\prime }R \end{array}$$

**Proof**:

**(1)**: For kernelized LSTD-RP, we just replace the basis set Φ*_n,D_* with *K_n,n_* in Equations (24) and (26), where *n* = *D*. Thus we get

(28) $$\begin{array}{lll} A_{\mathrm{CK}} & = & {\left({\mathrm{KC}}^{T}\right)}^{T}\;(I-\gamma P)\;({\mathrm{KC}}^{T})=\mathrm{CK}\;(K-\gamma K^{\prime })\;C^{T}\\ b_{\mathrm{CK}} & = & {\left({\mathrm{KC}}^{T}\right)}^{T}\;R=\mathrm{CKR} \end{array}$$

The value function *V̂_CK_* is,

(29) $$\begin{array}{lll} {\hat{V}}_{\mathrm{CK}}\;(s) & = & {\left(\mathrm{Ck}\;(s)\right)}^{T}\;w_{\mathrm{CK}}\\ & = & {\left(\mathrm{Ck}\;(s)\right)}^{T}\;A_{\mathrm{CK}}^{-1}\;b_{\mathrm{CK}}\\ & = & {\left(\mathrm{Ck}\;(s)\right)}^{T}\;{\left(\mathrm{CK}\;(K-\gamma K^{\prime })C^{T}\right)}^{-1}\;\mathrm{CKR} \end{array}$$

**(2)**:Next we develop the compressed version of kernelized value function approximation. As in [15], the kernelized value function *V̂_K_* can be represented as

(30) $${\hat{V}}_{K}\;(s)=k\;{\left(s\right)}^{T}\;{\left(I-\gamma K^{-1}\;K^{\prime }\right)}^{-1}\;K^{-1}\;R$$

According to the compressed kernel regression Equation (5), The compressed version is to make following change to Equation (30)

(31) $$\begin{array}{c} K\to \mathrm{CK}C^{T}\\ k\;(s)\to \mathrm{Ck}\;(s)\\ R\to \mathrm{CR} \end{array}$$

Also, we define

$$K_{C}=\mathrm{CK}C^{T},\;K_{C}^{\prime }={\mathrm{CK}}^{\prime }\;C^{T}$$

so we have

(32) $$\begin{array}{lll} {\hat{V}}_{\mathrm{KC}}\;(s) & = & {\left(\mathrm{Ck}\;(s)\right)}^{T}\;{\left(I-\gamma K_{C}^{-1}\;K_{C}^{\prime }\right)}^{-1}\;K_{C}^{-1}\;(\mathrm{CR})\\ & = & {\left(\mathrm{Ck}\;(s)\right)}^{T}\;{\left(K_{C}-\gamma K_{C}^{\prime }\right)}^{-1}\;(\mathrm{CR}) \end{array}$$

**(3)**: Based on the above analysis, we have

$$C^{T}\;C\approx I,\;K=K^{T}$$

so we have

(33) $$\begin{array}{lll} K_{C}\;K_{C} & = & \mathrm{CK}\;(C^{T}\;C)\;KC^{T}\approx \mathrm{CK}\;{\mathrm{KC}}^{T}\\ K_{C}\;K_{C}^{\prime } & & \mathrm{CK}\;(C^{T}\;C)\;K^{\prime }C^{T}\approx \mathrm{CK}K^{\prime }C^{T} \end{array}$$

Then the value function *V̂_KC_* is

(34) $$\begin{array}{lll} {\hat{V}}_{\mathrm{KC}}\;(s) & = & {\left(\mathrm{Ck}\;(s)\right)}^{T}\;{\left(I-\gamma K_{C}^{-1}\;K_{C}^{\prime }\right)}^{-1}\;K_{C}^{-1}\;(\mathrm{CR})\\ & = & {\left(\mathrm{Ck}\;(s)\right)}^{T}\;\underset{\mathrm{associate}}{\underset{︸}{{\left(I-\gamma K_{C}^{-1}K_{C}^{\prime }\right)}^{-1}\;K_{C}^{-1}\;K_{C}^{-1}}}\;\underset{\mathrm{associate}}{\underset{︸}{K_{C}\;(\mathrm{CR})}}\\ & = & {\left(\mathrm{Ck}\;(s)\right)}^{T}\;{\left(K_{C}\;K_{C}-\gamma K_{C}\;K_{C}^{\prime }\right)}^{-1}\;K_{C}(\mathrm{CR})\\ & \approx & {\left(\mathrm{Ck}\;(s)\right)}^{T}\;{\left(\mathrm{CK}\;(K-\gamma K^{\prime })\;C^{T}\right)}^{-1}\;(\mathrm{CKR})\\ & = & {\hat{V}}_{\mathrm{CK}}\;(s) \end{array}$$

Note that if we insert $K_{C}^{\prime -1}\;K_{C}^{\prime }$ instead of $K_{C}^{-1}\;K_{C}$ in the second equation, we would have

(35) $${\hat{V}}_{\mathrm{KC}}\;(s)\approx {\left(\mathrm{Ck}\;(s)\right)}^{T}\;{\left({\mathrm{CK}}^{\prime }\;(K-\gamma K^{\prime })\;C^{T}\right)}^{-1}\;({\mathrm{CK}}^{\prime }R)$$

So combining Equations (34) and (35) we can have Equation (27).

## 6. Experiment Result

### 6.1. Experimental Setting

We now present several comparison studies to show the effectiveness of our method. It is noteworthy to mention that the benefit of introducing Random Projections for feature compression lies mainly in reduction of computational cost. The overall performance, however, highly depends on the quality of the randomly generated compression matrix *C*; it is thus reasonable that the variance will be higher than in the ALD-based methods. Also we restrict the problems with finite sample set and large amount of features.

Unlike *L*_1_ regularization, LSPI-RP does not assume that the representation w.r.t the original basis is sparse. To implement *L*_1_-regularized method, we need the target function to be sparse in the selected representation. Mathematically, the sparsity appears in the bound instead of the dimension. So, if the signal is not sparse, its sparsity will be equal to the dimension, and thus, the large dimension appears in the bound. In LSPI-RP, however, instead of the requirement on sparsity of the original basis, a requirement is imposed on the features such that they are supposed to be of specific form in order to perform better than LSPI in high-dimensional space. So in LSPI-RP it would be of critical importance of the basis [2]. The second point, as mentioned above, the necessity of using LSPI-RP is as mentioned above on the scenarios where there is scarce amount of samples compared with the cardinality of features *F*, which would often lead to overfitting for regular LSPI. The setting is preferable for LSPI-RP where the number of samples is smaller than or at the same order of the number of features.

### 6.2. Experimental Analysis

First we define parameters used in the experiment:

- *d*: the compression dimension
- *n*: the number of samples, which is also the full dimension in kernelized LSPI
- *θ*: the compression ratio of *d/D*.
- *ɛ*: the threshold parameter for ALD dictionary.

#### Pendulum

**Figure 2.**
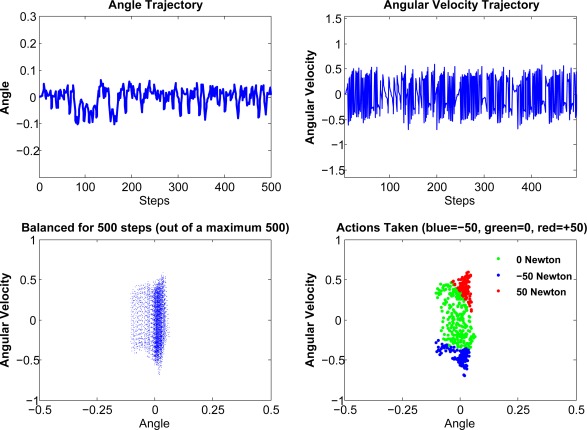
A Successful Run of Pendulum.

The pendulum domain is a typical under-actuated system which is highly unstable. Here we give some illustrative example of compressed kernelized LSPI in pendulum domain. Here we collect 2, 000 samples, and the compression ratio is 0.2. Here we take 20 runs on average, and the average runs to converge of compressed KLSPI, ALD-based KLSPI and regular LSPI are 7.4, 7.8, 8.6 runs. From here we can see that compressed KLSPI has the advantage of faster convergence. This merit will be more explicit as we will show in Figures 2 and 5.

#### Acrobot

**Figure 3.**
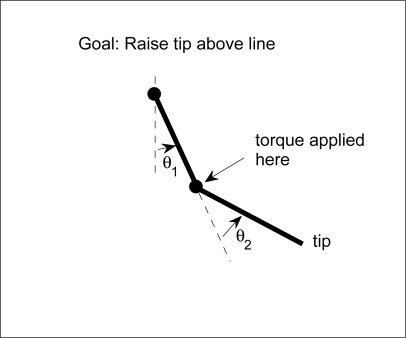
An Illustration of Acrobot.

**Figure 4.**
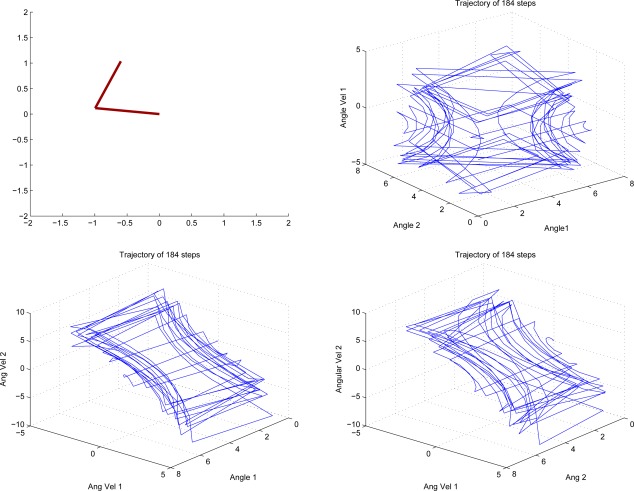
A Typical Trajectory of a Successful Swing-up of Acrobot.

The Acrobot is an under-actuated double pendulum which is a typical working benchmark for its nonlinear dynamics. It consists of two arms where torque can only be applied at the second joint. The system is described by four continuous variables: the two joint angles, *θ*_1_ and *θ*_2_, and the angular velocities, *θ̇*_1_ and *θ̇*_2_. There are three actions corresponding to positive (*a* = 1), negative (*a* = −1), and zero (*a* = 0) torque. The time step was set to 0.05 and actions were selected after every fourth update to the state variables according to the equations of motion. The goal for this domain is to raise the tip of the second link above a certain height in minimum time (we used a height of 1, where both links have a length of 1). The reward function is therefore −1 for each time step until the goal is achieved and the discount factor is *γ* = 0.99. Episodes begin with the all state variables at value 0 which corresponds to the two links hanging straight down and motionless. Figure 3 is an illustration figure of Acrobot. Figure 4 shows a snapshot of a successful run.

##### **(1)**: Comparison of Compression Ratio

In this experiment, we shall seek the optimal compressing dimension *d* with different number of samples. Up to our best knowledge, there is no tighter bound on how to choose *d* in compressed LSPI yet. In [2], a covering number bound of *d* is given with an empirical extension to a rule of thumb that $d=O(\sqrt{n})$. One can see that from the experimental results in Table 1, rule of thumb experience that $d=O(\sqrt{n})$ still applies.

##### **(2)**: Comparison of Kernel Sparsification: ALD *vs.* Compressed Sensing

In this experiment, several comparison studies are carried out to compare the two kernel sparsification technique: ALD and Compressed Sensing. Briefly, both two methods are highly adaptable and only depends on one parameter. The ALD method depends mainly on one parameter: the dictionary threshold *ε*. Likewise, the compressed sensing technique also depends on one parameter: the compressed ratio *θ* = *d/D*. To be fair, we will give identical compression dimension *d*, which means that the compressed dimension is equal to the size of the ALD dictionary. The problem setting in Experiment 2 is like this: given large amounts of data, the ALD dictionary is still considerably large. How to compress this dictionary further is an interesting topic worth attention. One heuristic idea, of course, is to make the accuracy threshold of ALD dictionary *υ* larger so that the accuracy tolerance becomes larger, and correspondingly the dictionary size becomes smaller. However, based on the compressive reinforcement learning framework, here is another attractive method, first we build the ALD dictionary with a very strict accuracy threshold *υ* and build an ALD dictionary with large size. Then we use the Random Projections to compress the basis generated by this “large” dictionary. Comparison studies are carried out and experimental results in Table 2 show that our method is better than the combo of these two methods, which in turn is better than purely using only one.

### 6.3. Compressed KLSPI in Value Function Approximation

**Figure 5.**
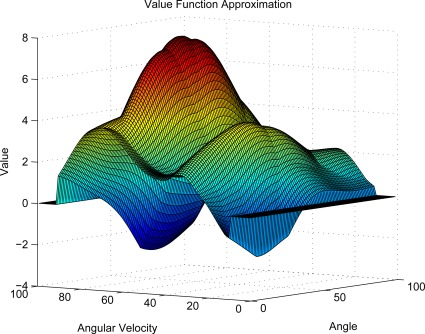
The Value Function of Pendulum.

In this experiment, we will show the approximated value function of Compressed Kernelized LSPI on pendulum domain. From Figure 5, we can see that although there are some dissimilarity, the approximated value function can produce good policy and is satisfactory enough to capture the main topology of the true value function.

### 6.4. Compare RBF Kernel and GGK Kernel

In here we give a new kernel function, namely, Geodesic Gaussian Kernel (GGK). Compared with RBF kernel, GGK can capture the topology on the manifold where the samples lies on instead of the Euclidean distance used in RBF kernel. Moreover, since GGKs are local by nature, the ill effects of local noise are constrained locally.

**Figure 6.**
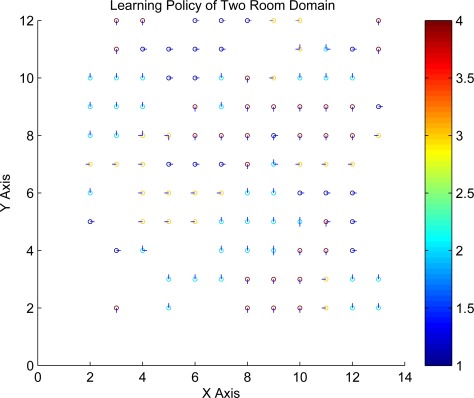
Learning Policy using GGK in Two Rooms Domain.

Geodesic Gaussian Kernels on Graphs:

A natural definition of the distance would be the shortest path. So we define Gaussian kernels on graphs based on the shortest path:

$$K\;(s,\;s^{\prime })=\text{exp}\;\left(-\frac{\mathrm{SP}\;{\left(s,\;s^{\prime }\right)}^{2}}{2\sigma^{2}}\right)$$

where *SP* (*s*, *s*′) denotes the shortest path from state *s* to state *s*′. Since the shortest path on the graph can be interpreted as a discrete approximation to the geodesic distance on a nonlinear manifold, geodesic Gaussian kernel (GGK) includes rich information of the manifold formed by the data. Shortest paths on graphs can be efficiently computed using the Dijkstra algorithm. The result for two-room domain using GGK is show in Figure 6.

## 7. Conclusions

In this paper a new nonparametric reinforcement learning framework called Compressive Kernelized Reinforcement Learning (CKRL) is proposed based on Gaussian process, compressed Sensing and random projections. We compare compressed kernelized LSPI, kernelized LSPI, and regular LSPI along with different kernels based on Euclidean distance and Graph-based Geodesic distance, respectively. Preliminary theoretical proof and experimental results are also given. There are various interesting future directions along this research venue. For instance, how to integrate *L*_1_ regularization into this framework is a promising topic to explore. Another promising future direction is to introduce this compressive reinforcement learning framework to policy gradient approaches with regular MDP [24] and POMDP [25].

## Figures and Tables

**Table 1.** Acrobot: Comparison of Compression Ratio.

Compression Ratio	Balancing Steps	With Failure?
100	412	Y
150	380	Y
300	312	N
400	227	N

**Table 2.** Comparison of Sparsification Technique: ALD and Random Projections.

ALD Threshold	Size of ALD Dic	Compression Ratio	Avg Blancing Steps
0.1	184	*	178
0.3	117	*	270
0.1	117	117*/*184	**194**
*	*	117/3, 000	403(with failure)
*	*	184/3, 000	341(with failure)
